# Synthesis, Optical, and Structural Studies of Iron Sulphide Nanoparticles and Iron Sulphide Hydroxyethyl Cellulose Nanocomposites from Bis-(Dithiocarbamato)Iron(II) Single-Source Precursors

**DOI:** 10.3390/nano8040187

**Published:** 2018-03-23

**Authors:** Athandwe M. Paca, Peter A. Ajibade

**Affiliations:** School of Chemistry and Physics, University of KwaZulu-Natal, Private Bag X01, Scottsville, Pietermaritzburg 3209, South Africa; 217076639@stu.ukzn.ac.za

**Keywords:** iron(II) dithiocarbamate, precursors, XRD, FeS nanoparticles, HEC nanocomposites

## Abstract

In this study, Fe(II) complexes of phenyldithiocarbamate, dimethyldithiocarbamate and imidazolyldithiocarbamate were used as single-source precursors to prepare iron sulphide nanoparticles by thermolysis in oleic acid/octadecylamine (ODA) at 180 °C. The nanoparticles were dispersed into hydroxyethyl cellulose (HEC) to prepare iron sulphide/HEC nanocomposites. Ultraviolet-Visible (UV-Vis), Photoluminescence (PL), Fourier Transform Infrared (FTIR), powder X-ray diffraction (pXRD), high-resolution transmission electron microscopy (HRTEM), Field emission scanning electron microscopy (FESEM), and energy dispersive X-ray spectroscopy (EDS) were used to characterize the iron sulphide nanoparticles and corresponding HEC nanocomposites. The absorption spectra studies revealed that the nanoparticles were blue shifted due to quantum confinement and the optical band gaps of the nanoparticles are 4.85 eV for FeS1, 4.36 eV for FeS2, and 4.77 eV for FeS3. The emission maxima are red-shifted and broader for the nanoparticles prepared from phenyldithiocarbamate. Rod-like and spherically shaped iron sulphide particles were observed from the HRTEM images. The crystallite sizes from the HRTEM images are 23.90–38.89 nm for FeS1, 4.50–10.50 nm for FeS2, and 6.05–6.19 nm for FeS3 iron sulphide nanoparticles, respectively. pXRD diffraction patterns confirmed that FeS1 is in the pyrrhotite-4M crystalline phase, FeS2 is in the pyrrhotite phase, and FeS3 is in the troilite phase of iron sulphide. The phases of the iron sulphide nanoparticles indicate that the nature of the precursor complex affects the obtained crystalline phase. FTIR spectra studies confirmed the incorporation of the nanoparticles in the HEC matrix by the slight shift of the O–H and C–O bonds and the intense peaks on the nanoparticles. FESEM images of the iron sulphide nanoparticles showed flake-like or leaf-like morphologies with some hollow spheres. The EDS confirmed the formation of iron sulphide nanoparticles by showing the peaks of Fe and S.

## 1. Introduction

In recent years, metallic nanoparticles have received tremendous research attention because of their unique physical, chemical and biological properties associated with their broad surface-to-volume ratio and due to quantum confinement effects [1,2,3,4,5,6]. These properties make them useful for the development of novel applications in communications, energy storage, data storage, optic sensing, transmission, environment protection, cosmetics, biology, and medicine [7]. It is envisaged that nanoparticles will play significant roles in medicine in the near future, because they offer a therapeutic and diagnostic potential that is almost impossible to achieve using bulk materials [8]. Nanoparticles can be used in drug delivery systems for a variety of medicines, since they exist in the same scale as proteins [8,9] and their unique properties attract interest in the on-going development of cancer treatment [10]. As particles get smaller, more atoms or molecules constituting the particle are exposed on the surface of the particle. This plays a great role in influencing the bonding of the particle with other materials [11].

The single-source precursor (SSP) approach has become a versatile and effective route for the preparation of metal sulphide nanoparticles [12,13,14,15]. This approach allows controlled growth of nanocrystals with excellent optical properties [16]. The precursor should be able to decompose easily to produce pure metal sulphide nanoparticles and volatile and unreactive by-products to avoid any contamination of the desired product [17]. The single-source precursors replaced poisonous organometallic compounds [18] and produced improved crystalline monodispersed semiconductor nanoparticles [19,20]. Trindade et al. reported thermolyzation of SSP based on metal dithiocarbamate complexes for the synthesis of CdS and CdSe nanoparticles. The choice of SSP with metal-chalcogenide bonds offers a suitable reactive intermediate for the preparation of nanoparticles at high temperatures, allowing for the synthesis of nanomaterials from relatively nontoxic precursor complexes [20,21]. Ajibade et al. investigated group 12 dithiocarbamate complexes to prepare metal sulphide nanoparticles using this method [22]. The SSP method uses high boiling point coordinating solvents such as trioctylphosphineoxide (TOPO) [20], hexadecylamine (HDA) [23,24], octadecylamine (ODA), or oleylamine [25,26] as capping agents and requires an inert environment. The capping agent hinders the growth of nanoparticles and stabilizes them from aggregation to give rise to the modified structural, morphological, and optical properties of the nanoparticles [27]. The single source precursor decomposes in hot capping agent to produce moisture stable nanoparticles free of defects and impurities from by-products [23,28].

Most of the work on the use of single-source precursors for the preparation of nanomaterials has focused on heavy and toxic transition metal ions [12,13,14,19,20,21,22,23] that cannot be used for biomedical application. There is a need to develop single source precursors from metal ion that are biocompatible such as iron [24,26,27,28]—hence the current study focuses on the use of iron(II) dithiocarbamate complexes as precursors for the preparation of iron sulphide nanoparticles. Different forms of iron sulphides (Fe*_n_*S*_m_*) exist in nature with an Fe:S ratio of 0.1–1.05 [29]. The stoichiometric ratio between Fe and S in iron sulphides and their crystalline structures gives them some magnetic and electrical properties [29]. Within the crystalline structure of iron sulphide, there are seven different phases: Greigite, troilite, pyrrhotite, iron sulphide, mackinawite, marcasite, and pyrite [27,30,31]. The synthesis of iron sulphides is a bit challenging compared to the synthesis of iron oxide due to the coexistence of a strong reducible ferric ion and oxidizable sulphides ion in iron sulphides [29]. Phase control is of paramount importance during the synthesis of iron sulphide, since the performance of each system depends on it [27]. In this study, we present the optical and structural studies of iron sulphide nanoparticles in three different crystalline phases prepared from three bis-(dithiocarbamato)iron(II) complexes single-source precursors. The iron sulphide nanoparticles were further dispersed into hydroxyethyl cellulose (HEC) to prepare iron sulphide/HEC nanocomposites. The optical and structural properties of the iron sulphide nanoparticles/HEC nanocomposites were studied using Ultraviolet-Visible (UV-Vis), Photoluminescence (PL), powder X-ray diffraction (pXRD) high-resolution transmission electron microscopy (HRTEM), Field emission scanning electron microscopy (FESEM), energy dispersive X-ray spectroscopy (EDS), and Fourier Transform Infrared Spectroscopy (FTIR).

## 2. Materials and Methods

### 2.1. Materials and Reagents

Iron(II) acetate (Fe(CO_2_CH_3_)_2_), ethanol, octadecylamine (ODA), oleic acid, hydroxyethyl cellulose (HEC) [CAS 9004-62-0, pH (10 g/L, H_2_O, 20 °C)], tetrahydrofuran (THF), and methanol were purchased from Merck (Darmstadt, Germany) and used as provided without any purification. Potassium salt of phenyldithiocarbamate, dimethyldithiocarbamate, and imidazolyl dithiocarbamate ligands were prepared using modified literature method [32].

### 2.2. Synthesis of Bis-(Dithiocarbamato)Iron(II) Complexes

Ethanol solution of Fe(CO_2_CH_3_)_2_ (10 mmol, 1.74 g) was added to an ethanol solution of either potassium phenyl dithiocarbamate (20 mmol, 4.15 g), potassium dimethyldithiocarbamate (20 mmol, 3.19 g), or potassium imidazolyl dithiocarbamate (20 mmol 3.65 g) with stirring at room temperature for 2 h until precipitation was completed. The precipitate was filtered, washed with cold ethanol, and dried under vacuum. The complex from each ligand is formulated as: [Fe(S_2_CNHC_6_H_5_)_2_], [Fe(S_2_CN(Me)_2_)_2_], and [Fe(S_2_CNC_3_H_3_N)_2_].

### 2.3. Preparation of Iron Sulphide Nanoparticles

Octadecylamine (10 g) placed in a three-necked flask, equipped with a reflux condenser, thermometer, and a rubber septum was heated to 180 °C with vigorous stirring under nitrogen. The precursor (0.5 g) dispersed in oleic acid (6 mL) was injected into the hot solution using a syringe. A decrease in temperature of about 15–30 °C was observed. The reaction was maintained at 180 °C for 1 h. The content on the flask was cooled to approximately 70 °C, an excess amount of cold ethanol was added, and the formed nanoparticles were isolated by centrifugation at 2000 rpm for 30 min. The nanoparticles were washed several times with cold ethanol to remove any excess ODA and oleic acid, and were left to dry under vacuum. The prepared nanoparticles were labelled FeS1, FeS2, and FeS3 prepared from [Fe(S_2_CNHC_6_H_5_)_2_], [Fe(S_2_CN(Me)_2_)_2_], and [Fe(S_2_CNC_3_H_3_N)_2_], respectively.

### 2.4. Preparation of Iron Sulphide Nanoparticles/hydroxyethylcellulose (HEC) Nanocomposites

The iron sulphide HEC nanocomposites were prepared by the solution casting method [33]. HEC (1.0 g) was dissolved in distilled water (20 mL) and stirred for 2 h. To the viscous polymer solution, iron sulphide nanoparticles (0.05 g) dissolved in THF were added. The mixture was stirred vigorously for 1 h at 60 °C. The solution was poured into a petri-dish, and the solvent was evaporated under vacuum. 

### 2.5. Characterization Techniques

The FTIR spectra were recorded on Perkin Elmer Spectrum 100 FTIR spectrometer (4000–650 cm^−1^) (Waltham, MA, USA). Absorption spectra of nanoparticles were measured in chloroform, using a Perkin Elmer Lambda 25 spectrophotometer (Waltham, MA, USA) at room temperature. Emission spectra of the particles were recorded on a Perkin Elmer LS 45 fluorescence spectrometer (Waltham, MA, USA), and the data was collected at room temperature. The pXRD analysis of the samples was recorded on a Bruker D8 advanced diffractometer (Billerica, MA, USA) using Cu Kα radiation. Samples were loaded on flat steel and scanned from 5° and 85°. The HRTEM images were obtained by a JEOL JEM-2100 electron microscope (Akishima, Tokyo, Japan). FESEM images were obtained by ZEISS FEGSEM Ultra plus (Oberkochen, Germany).

## 3. Results and Discussion

### 3.1. Powder X-ray Diffraction (pXRD) Studies of FeS1, FeS2, and FeS3 Iron Sulphide Nanoparticles

**Figure 1:**
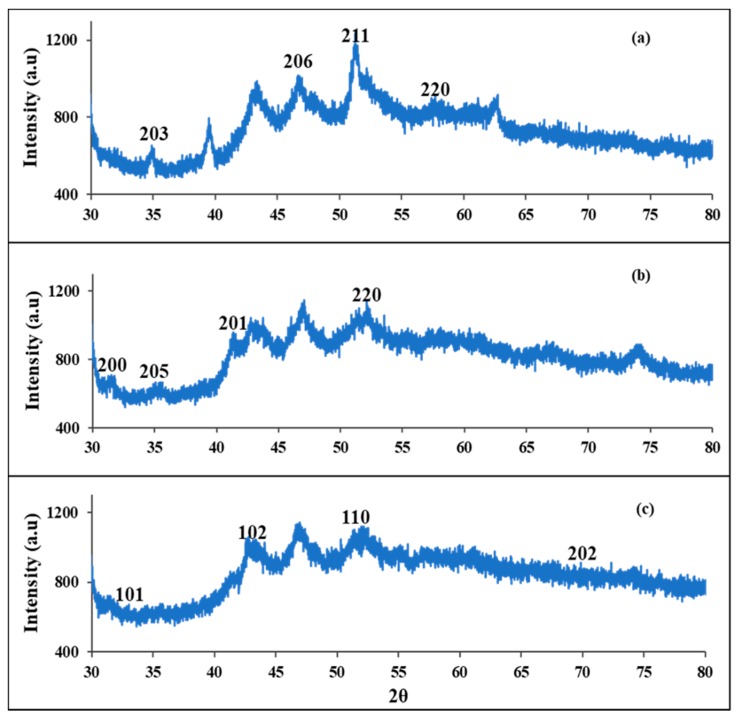
Powder XRD patterns of FeS1 (**a**); FeS2 (**b**) and FeS3 (**c**) iron sulphide nanoparticles from various Fe(II) dithiocarbamate complex at 180 °C.

The powder X-ray diffraction (pXRD) patterns of the FeS1, FeS2, and FeS3 iron sulphide nanoparticles prepared from iron(II) dithiocarbamate complexes at 180 °C are presented in Figure 1. The X-ray diffraction patterns of FeS1 showed about eight diffraction peaks at 2*θ* equals 34.9°, 46.9°, 51.5°, and 58.0° corresponding to (203), (206), (211), and (220). All the major diffraction peaks can be indexed to the standard diffraction data of the iron sulphide pyrrhotite-4M crystalline phase with chemical formula Fe_7_S_8_ (International Centre for Diffraction Data (ICDD) ref code: 00-029-0723) [20]. The XRD pattern of FeS2 shown in Figure 1b showed reflections at peaks position at 2*θ* equals 31.1°, 35.0°, 41.6°, and 52.8°, which can be indexed to (200), (205), (201), and (220) planes of pyrrhotite iron sulphide crystalline phase with chemical formula Fe_9_S_10_ (ICDD ref code: 04-020-0793) [34]. The XRD patterns of FeS3 nanoparticles presented in Figure 1c showed peaks at 2*θ* equals 32.1°, 43.1°, 51.8°, and 70.2°. These peaks correspond to (101), (102), (110), and (202) crystallographic planes of troilite (FeS) iron sulphide nanoparticles with card number (ICDD ref code: 01-084-3944) [35].

### 3.2. Morphological Studies of the FeS1, FeS2, and FeS3 Iron Sulphide Nanoparticles

**Figure 2:**
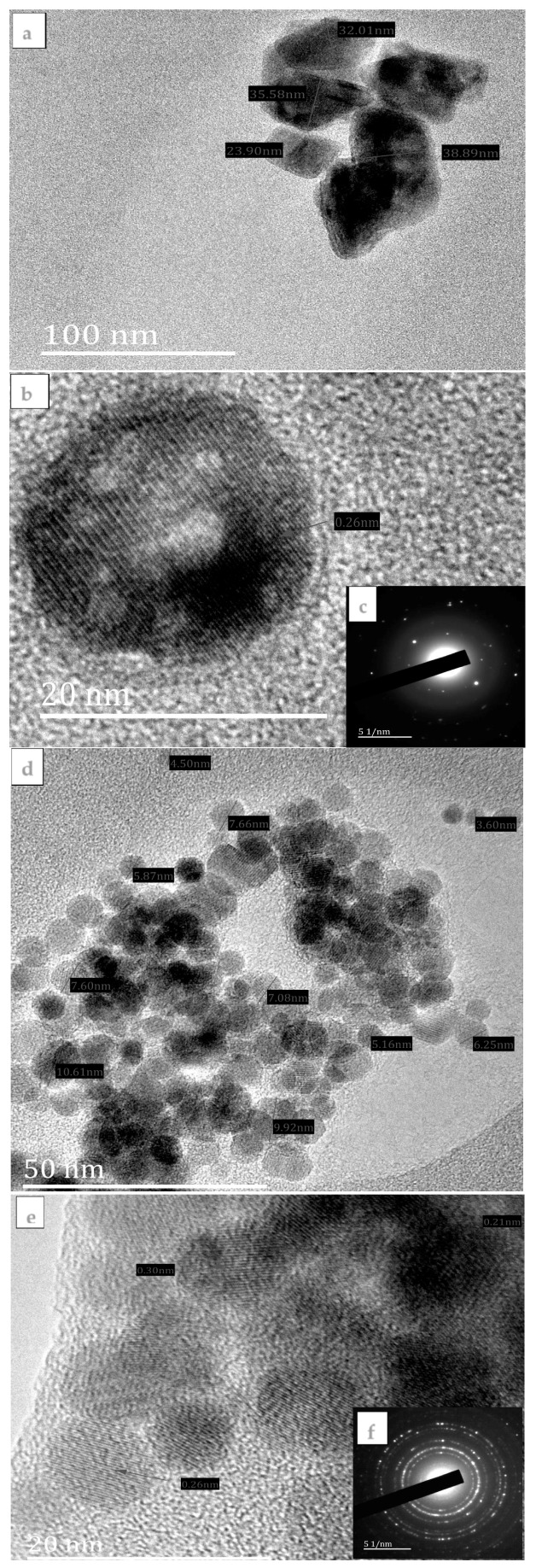

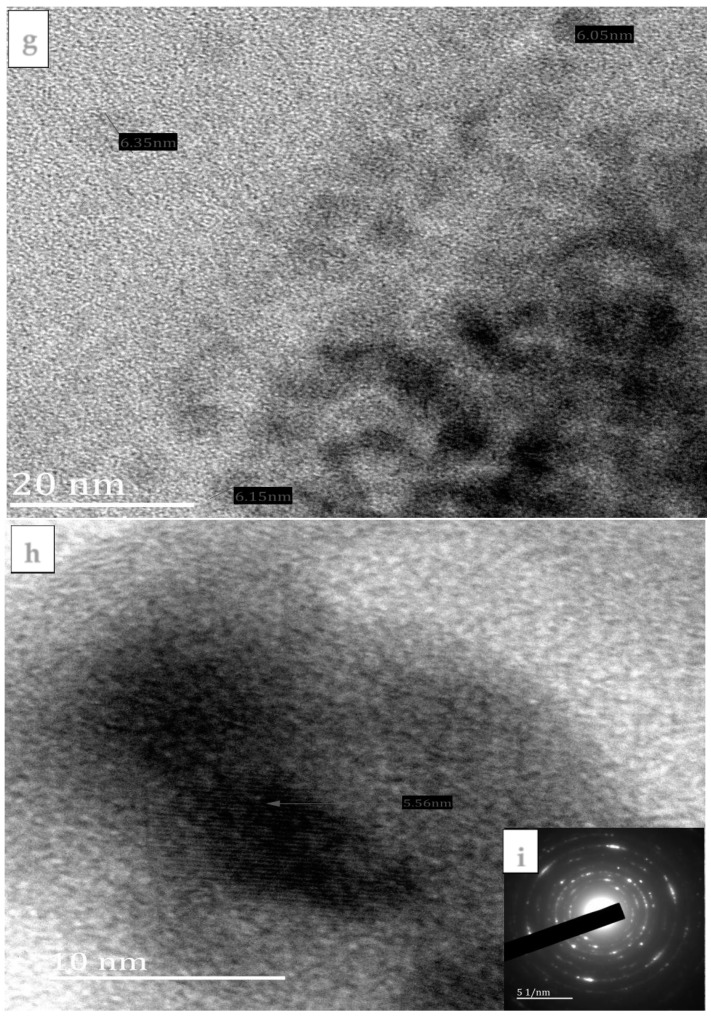
HRTEM image (**a**) lattice fringe (**b**) and SAED pattern (**c**) of FeS1 nanoparticles; HRTEM image (**d**) lattice fringe (**e**) and SAED pattern (**f**) of FeS2 nanoparticles; HRTEM image (**g**) lattice fringe (**h**) and SAED pattern (**i**) of FeS3 nanoparticles.

The HRTEM images, lattice fringes, and SAED patterns of FeS1, FeS2, and FeS3 iron sulphide nanoparticles are presented in Figure 2. HRTEM image of FeS1 shown in Figure 2a shows large, spherically shaped iron sulphide nanoparticles with crystallite sizes in the range 23.90–38.89 nm. The nanoparticles are irregular in shape and appeared aggregated. The lattice fringes of FeS1 shown in Figure 2b revealed polycrystalline nanoparticles consisting of many crystal grains. The interplanar distances is 0.26 nm. The SAED patterns of FeS1 are shown in Figure 2c and bright spots forming ring patterns confirm that the iron sulphide nanoparticles are crystalline in nature. There are also some diffused rings in the SAED patterns ascribed to the amorphous octadecylamine-capping agent.

The HRTEM image of FeS2 (Figure 2d) shows iron sulphide nanoparticles that are almost spherical in shape. The particle sizes for FeS1 nanoparticles are in the range 4.50–10.60 nm with some agglomerated particles. The lattice fringe of FeS2 (Figure 2e) revealed that the iron sulphide nanoparticles consist of extremely small crystals, indicating that FeS2 nanoparticles are polycrystalline. In the small crystal grains, three sets of interplanar distances, 0.21, 0.26, and 0.29 nm, dominated. Figure 2f shows the SAED pattern of FeS2 showing small spots that form rings ascribed to the Bragg reflections from the individual crystallite of the iron sulphide nanoparticles. Figure 2g shows the HRTEM image of FeS3 with crystallite sizes, which ranges between 6.05 and 6.19 nm. The FeS3 iron sulphide nanoparticles appeared as aggregates of small quantum dots with no distinct shapes but some aggregated into rod-like patterns. Figure 2h shows the lattice fringe of FeS3 nanoparticles. The SAED patterns of FeS3 Figure 2i shows diffuse rings with some bright spots indicating that the nanoparticles consist of mixtures of crystalline iron sulphide nanoparticles and some amorphous materials which might be due to the capping agent most probably in excess around the nanoparticles.

**Figure 3:**
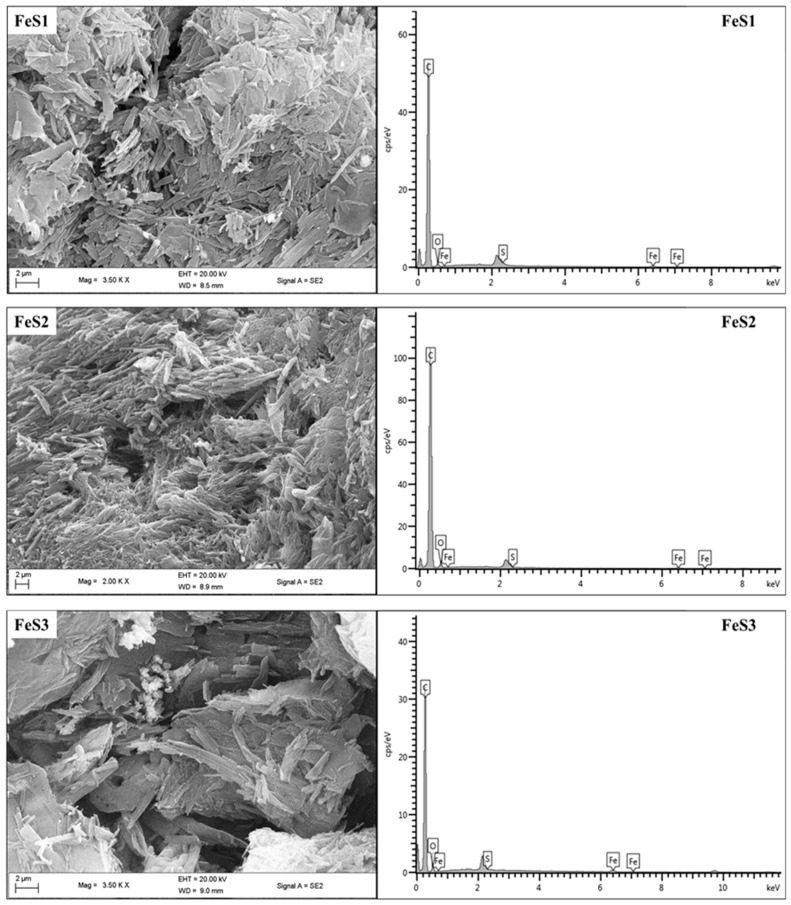
FESEM/EDS micrographs of FeS1, FeS2, and FeS3 iron sulphide nanoparticles.

FESEM images and EDS spectra of FeS1, FeS2, and FeS3 iron sulphide nanoparticles are shown in Figure 3. FESEM images of FeS1 and FeS2 nanoparticles show very similar clustered leaf-like morphologies with no apparent patterns. The EDS spectra showed peculiar Fe and S peaks, which indicate the presence of the iron sulphide nanoparticles. FESEM image of FeS3 nanoparticles shows layered rough surfaces with white patches ascribed to the excess capping agent. The presence of the Fe and S peaks in the EDS spectrum confirms the successful preparation of the iron sulphide nanoparticles.

### 3.3. Optical Studies of FeS1, FeS2, and FeS3 Iron Sulphide Nanoparticles

**Figure 4:**
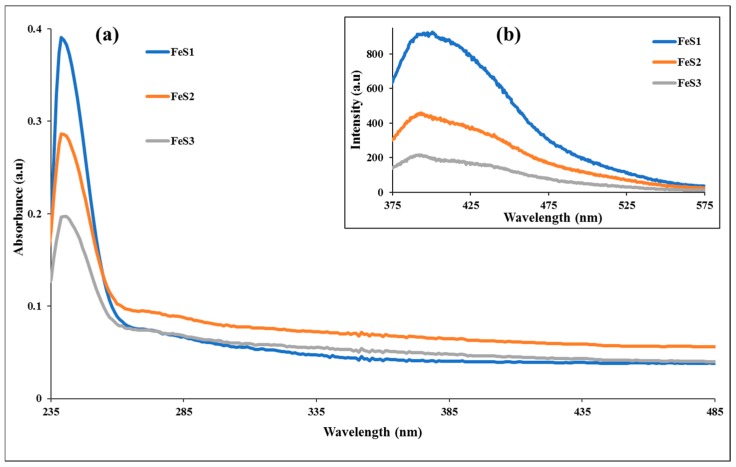
Absorption (**a**) and emission (**b**) spectra of the FeS1, FeS2, and FeS3 iron sulphide nanoparticles from different Fe(II) dithiocarbamate complexes thermolyzed at 180 °C.

**Figure 5:**
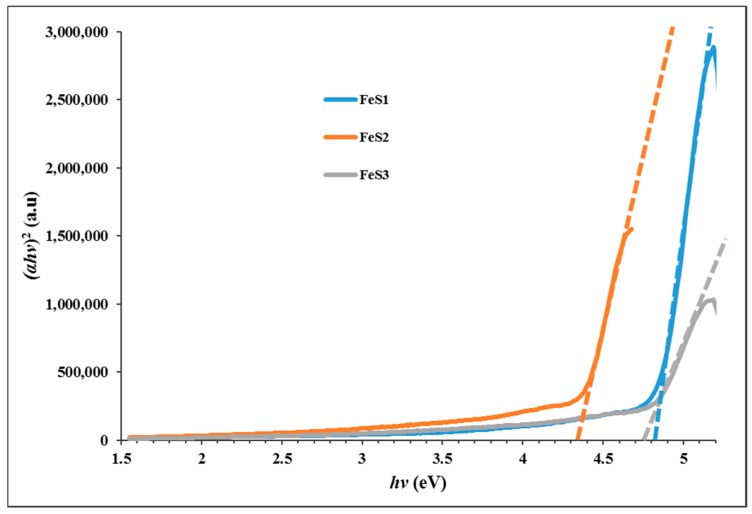
Tauc plot of the FeS1, FeS2, and FeS3 iron sulphide nanoparticles from different Fe(II) dithiocarbamate complexes at 180 °C.

The absorption spectra of the as-prepared iron sulphide nanoparticles are presented in Figure 4a, and they show absorption band edges at 240, 241, and 242 nm, respectively, for FeS1, FeS2, and FeS3 nanoparticles. These bands are blue shifted in comparison to bulk materials, and this could be ascribed to the as-prepared iron sulphide nanoparticles exhibiting strong quantum confinement effects that are attributed to the small crystallite sizes of the iron sulphide nanoparticles [36]. The emission spectra of the FeS1, FeS2, and FeS3 nanoparticles are shown in Figure 4b. The emission spectrum of FeS1 obtained from bis-(phenyldithiocarbamato)iron(II) is far broader than FeS2 and FeS3. This indicates that the phenyl ring of the dithiocarbamate ligand plays some role in the decomposition and thus affects the optical properties of the resulting nanoparticles. The emission spectra showed broad emission curves with emission maxima at 400 nm for FeS1, 398 nm for FeS2, and 394 nm for FeS3 iron sulphide nanoparticles. The emission maxima of the iron sulphide nanoparticles are red-shifted in comparison to the absorption band edges [37]. The optical band gap of the iron sulphide nanoparticles were determined from the Tauc plot shown in Figure 5. These were found to be 4.85 eV for FeS1, 4.36 eV for FeS2, and 4.77 eV for FeS3. 

### 3.4. FTIR Spectra and FESEM Structural Studies of FeS1, FeS2, and FeS3 Iron Sulphide/HEC Nanocomposites

**Figure 6:**
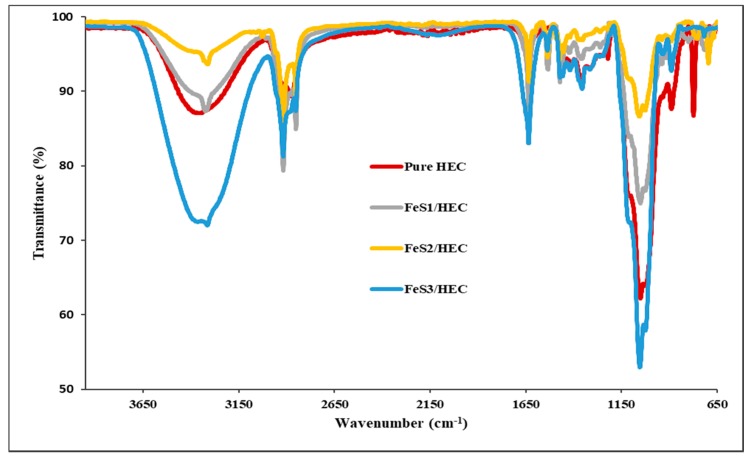
FTIR spectra of pure HEC and iron sulphide/HEC nanocomposites prepared from FeS1, FeS2, and FeS3 nanoparticles.

FTIR spectroscopy was used to study the interactions between the reactive hydroxyl groups and alkoxyl groups on hydroxyethyl cellulose (HEC) chains. FTIR spectra of the pure HEC and iron sulphide/HEC nanocomposites are presented in Figure 6. The pure HEC spectrum shows characteristic bands at 3343, 2872, 1635, and 1055 cm^−1^ assigned to the v(O–H), v(C–H), v(C=O), and v(C–O) stretching vibrations. The iron sulphide/HEC nanocomposites show similar bands at the same frequencies but with lower intensities, which confirms the interactions of the HEC and the iron sulphides nanoparticles except for FeS2/HEC nanocomposites, which suggests that nanoparticles are very small. This observation confirms the interaction of the hydroxyl and alkoxyl groups of the HEC polymer with the iron sulphide nanoparticles. Moreover, nanoparticles with large particle sizes are more stabilized, indicating that the larger particles interact more with the HEC matrix than the smaller ones; hence, the v(O–H) intensity is lower.

**Figure 7:**
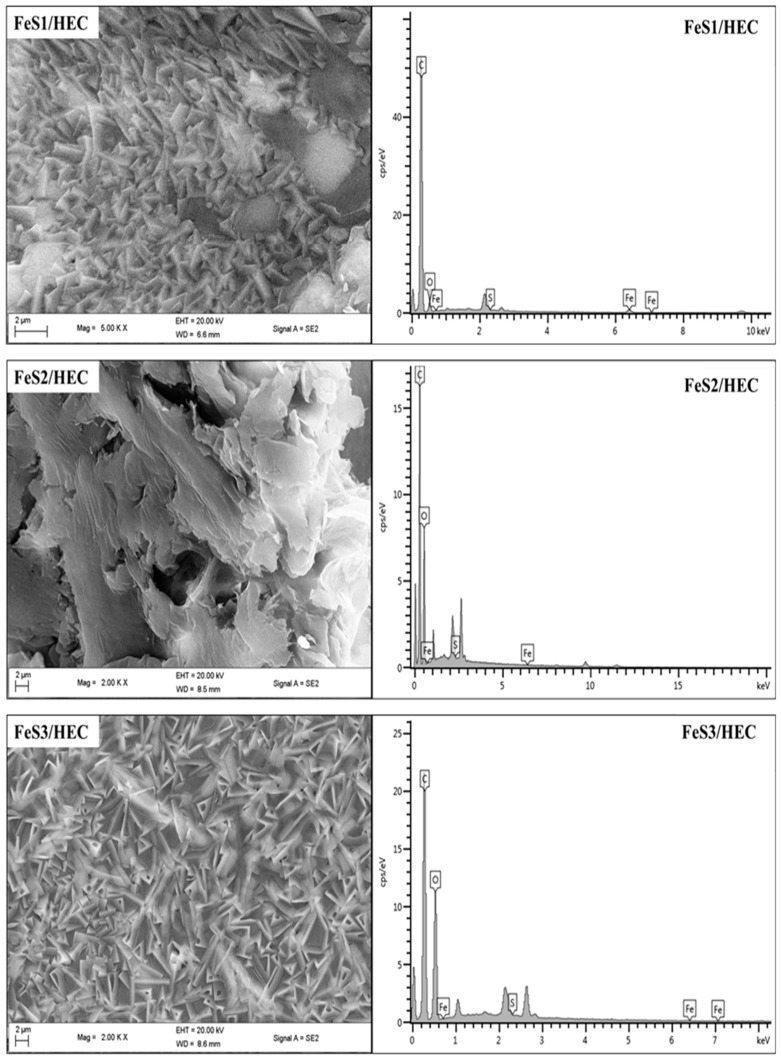
FESEM/EDS micrographs of iron sulphide/HEC nanocomposites prepared from FeS1, FeS2, and FeS3 nanoparticles.

FESEM images and EDS spectra of nanocomposites prepared from iron sulphide are presented in Figure 7. The FESEM image of FeS1/HEC nanocomposites shows flake-like morphology that appeared white at higher magnification. The arrangement of the flakes does not follow any particular pattern. The EDS confirms the presence of the iron sulphide in the HEC matrix in the iron sulphide/HEC nanocomposites. The FESEM image of FeS2/HEC nanocomposites shows no distinct morphology, but the surface appeared with almost threadlike patterns and some hollow spheres. One side of the surface appeared almost white, while most of the nanocomposites surfaces appeared dark. The EDS confirmed the incorporation of the iron sulphide nanoparticles into the HEC matrix. The FESEM image of FeS3/HEC shows surface morphologies that are leaf/flake-like, with some of them containing triangular hollow spheres. The surface morphology of these particular nanocomposites does not contain any white patches, unlike the other two, and the nano-flakes are arranged in regular patterns [38]. EDS confirmed the presence of the iron sulphide nanoparticles in the HEC matrix. The differences observed in the surface morphologies of the three nanocomposites are an indication that the characteristic properties of the iron sulphide nanoparticles, such as their sizes and shapes, affect their interactions with the HEC polymer leading to the differences in the appearance of the resultant iron sulphide/HEC nanocomposites.

## 4. Conclusions

We report the synthesis and structural studies of three iron sulphides nanoparticles prepared from three bis-(dithiocarbamato)iron(II) complexes thermolysed at 180 °C in oleic acid and octadecylamine. The absorption spectra of the nanoparticles indicate that they are blue shifted, while the emission maxima are red shifted. The intensity of the absorption and emission maxima is dependent on the nature of the precursor complex. It follows the order phenyl > alkyl > diazole. The optical band gaps of the nanoparticles are 4.85 eV for FeS1, 4.36 eV for FeS2, and 4.77 eV for FeS3. Powder X-ray diffraction patterns confirm that the iron sulphide nanoparticles are in three different crystalline phases of the iron sulphides with FeS1 in the pyrrhotite-4M crystalline phase, FeS2 in the pyrrhotite phase, and FeS1 is in the troilite phase of iron sulphide. These results indicated that the single source precursor used for the preparation of the nanoparticles has effects on the resultant crystalline phase of the iron sulphide nanoparticles. HRTEM images showed that iron sulphide nanoparticles shapes varied from spherical to rod-like with sizes in the range 23.90–38.89 nm for FeS1, 4.50–10.50 nm for FeS2, and 6.05–6.19 nm for FeS3, respectively. The crystallite sizes of the iron sulphide nanoparticles shows that nanoparticles obtained from precursors containing a phenyl ring are generally bigger than those with the methyl group. These results indicate that the ease of decomposition of the precursor complex determine the size of the nanoparticles. The FESEM images of the iron sulphide nanoparticles showed flake-like or leaf-like morphologies with some hollow spheres. The EDS confirmed the formation of iron sulphide nanoparticles with Fe and S peaks. FTIR spectra studies indicate the iron sulphide/HEC nanocomposites show similar bands at the same frequencies but with lower intensities, which confirms the interactions of the HEC polymer and the iron sulphides nanoparticles.
